# Post‐operative incidence of lymphedema after RARP with or without extended pelvic lymph node dissection in a cohort study

**DOI:** 10.1002/bco2.466

**Published:** 2024-12-18

**Authors:** Andries Clinckaert, Laura Ysenbaardt, Annabel Bijnens, Charlotte Van Calster, Inge Geraerts, Steven Joniau, Nele Devoogdt, Luc Bijnens, Wouter Everaerts

**Affiliations:** ^1^ Department of Cellular and Molecular Medicine KU Leuven Leuven Belgium; ^2^ Department of Urology University Hospitals of Leuven Leuven Belgium; ^3^ Department of Rehabilitation Sciences University of Leuven Leuven Belgium; ^4^ Data science institute UHasselt Hasselt Belgium

**Keywords:** extended pelvic lymph node dissection, lymphedema, robot‐assisted radical prostatectomy

## Abstract

**Objectives:**

Lymphedema of the lower limbs and pubic area is a potential complication following extended pelvic lymph node dissection (ePLND) during robot‐assisted radical prostatectomy (RARP). The incidence of lymphedema after ePLND has not been systematically reported in the literature. This study aimed to determine the incidence of lymphedema, describe its clinical characteristics and identify specific risk factors in patients undergoing RARP with or without ePLND.

**Methods:**

A retrospective cohort study was conducted at a tertiary referral centre between April 2016 and July 2020. Structured electronic case report forms (eCRFs) integrated into the electronic health record system were used to document intraoperative, perioperative and postoperative data. The primary endpoint was the incidence of lymphedema. Secondary endpoints included risk factors for and localization of the postoperative lymphedema.

**Results:**

A total of 500 patients who underwent RARP were included, with 301 patients undergoing ePLND and 199 patients without any form of PLND. Median follow‐up period was 18 (range 3–49) months. Seventy‐eight out of 301 (26%) of patients who underwent ePLND developed lymphedema, compared to only 2 out of 199 (1%) patients without ePLND. In most patients (49/301, 16%), lymphedema was mild (grade 1), whereas 29 patients (10%) developed grade 2 lymphedema. Twenty‐six patients (9%) received decongestive lymphatic therapy. The most frequent site of lymphedema occurrence were the lower (54%) and the upper legs (40%). The number of nodes removed during RARP was identified as a risk factor for post‐operative lymphedema (OR 1.04; *p* < 0.05).

**Conclusions:**

In this cohort study, approximately one in four patients undergoing RARP with ePLND developed lower limb and/or midline oedema, whereas one in ten patients started decongestive lymphatic therapy for symptomatic lymphedema. These findings provide valuable information for patient counselling about the potential benefits and risks of ePLND.

## 1 INTRODUCTION

Performing an extended pelvic lymph node dissection (ePLND) in patients undergoing a radical robot‐assisted prostatectomy (RARP) is considered the best staging procedure for determining pelvic lymph node metastasis.^1^ Nevertheless, the therapeutic benefit of performing a PLND remains debated.^2^ Moreover, this procedure is associated with significant postoperative morbidity, including lymphoceles, thromboembolic events and lymphedema of the legs, the genital and suprapubic region.^2^

Lymphedema after ePLND results from damage to the lymphatic vessels draining the lower limb and pubic region, resulting in chronic swelling, erythema and sensation of heaviness. Stages of limb lymphedema have been described by the International Society of Lymphology, ranging from subclinical oedema (stage 0), oedema subsiding with limb elevation (stage 1), oedema persisting upon limb elevation (stage 2) to lymphatic elephantiasis (Stage 3).^3^

Patients suffering from secondary lymphedema after ePLND are treated with decongestive lymphatic therapy, including compression bandages/stocking, skin can and muscle exercises. Although this therapy improves the symptoms of lymphedema, especially when initiated at an early stage, it does not cure the lymphatic obstruction and patients may need lifelong treatment.^4^

Despite its potential clinical impact, the incidence of secondary lymphedema in prostate cancer patients undergoing pelvic lymph node dissection is poorly understood.^5^ Lack of standardized reporting of lymphedema as a complication of pelvic lymph node dissection has resulted in a wide range of lymphedema rates varying from 0 to 14% in men undergoing PLND and from 18 to 29% in men undergoing staging PLND followed by pelvic nodal irradiaton.^5^, ^6^, ^7^

Age, obesity, lymph node status, venous obstruction, extent of the surgery and adjuvant treatments have been identified as risk factors for developing lymphedema after oncological surgery.^8^, ^9^ In prostate cancer, however, no risk factors for postoperative lymphedema have been identified. Thus, there is a need for a more comprehensive understanding of the incidence of secondary lymphedema, its clinical characteristics and the associated risk factors.

In this cohort study, we estimate the incidence of lymphedema in prostate cancer patients undergoing RARP with ePLND in our centre and determine if known risk factors for postoperative lymphedema in other malignancies also apply to prostate cancer surgery.

## 2 PATIENTS AND METHODS

### 2.1 Inclusion population and data collection

This study was approved by the local Ethics Committee. Data from all patients who underwent robot‐assisted radical prostatectomy with or without PLND in our tertiary referral centre between 4 April 2016 and 6 July 2020 were prospectively collected in a structured database. Exclusion criteria included retropubic radical prostatectomy, a follow‐up time less than 3 months and metastatic disease. After excluding 240 patients, our population consisted of 500 unique patients. Main reasons for exclusion were open radical prostatectomy and insufficient (<3 months) follow up at our centre.

Intraoperative, peri‐ and postoperative data were prospectively collected using structured electronic case report forms (eCRFs) integrated in the electronic health record system. These eCRFs are integrated in the clinical routine of our care pathways.^10^ The eCRFs contain specific items on the presence of lymphedema (yes/no), the location of lymphedema (left/right; upper leg/lower leg/ft; scrotal or penile oedema) and the treatment (conversative/compression stockings). Follow‐up visits were scheduled according to the hospital's protocol at the following intervals: before surgery, 4–6 weeks, at 3–6 months,12 months and 24 months after surgery. Data not collected through the eCRF forms were manually extracted from the electronic medical records.

### 2.2 Surgical procedure

All procedures were performed by, or under direct supervision of two experienced robotic surgeons. The pelvic lymph node dissection was performed before initiating the prostatectomy. The eLND template included all lymphatic tissue overlying the external iliac vessels, the obturator fossa and the internal iliac artery. The borders of this template consisted of the perivesical fat medially, the genitofemoral nerve laterally, the ureteral crossing of the iliac bifurcation superiorly and the pubic bone inferiorly. Bipolar energy was used to coagulate lymph vessels, without the routine used of clips. Pneurmoperitoneum was maintained at 12 mmHg during the procedure. The peritoneum was left open after completion of the prostatectomy, but no peritoneal fixation of interposition was performed. A surgical drain was not routinely placed. Patients were mobilized postoperatively on the day of surgery. Patients in the ePLND group were given compression stocking until catheter removal (day 7) and low molecular weight heparin for 30 days.

### 2.3 Endpoints

The primary endpoint was to determine the post‐operative incidence of lymphedema after RARP with ePLND. Lymphedema was defined as a self‐reported swelling of at least one of the predefined lymphedema locations (upper leg, lower leg, scrotum or suprapubic region) persisting beyond 6 weeks post‐operatively and was confirmed on clinical examination by one of the treating healthcare workers (urologist or prostate cancer nurse‐specialist). If lower limb lymphedema was established at one point after surgery, we recorded the grade of lymphedema using the International Society of Lymphology (ISL) scale, the location of the swelling and whether the patient was referred to a specialized lymphedema clinic.^3^

We evaluated potential risk factors for secondary lymphedema. Potential risk factors were identified from literature on secondary lymphedema in breast and other gynaecological cancers.^11^, ^12^, ^13^ Preoperative body mass index (BMI), number of nodes removed during surgery and adjuvant or salvage radiotherapy were investigated as possible risk factors.^11^, ^12^, ^13^Additionally, based on expert input we also evaluated pre‐ or perioperative inguinal hernia repair as a risk factor.

### 2.4 Statistics

The data collected for this study were summarized and explored using statistical models with the statistical software R studio based on R version 3.6.0. Demographic and post‐intervention characteristics were summarized in tables. The choice of summarization to mean or median for continuous variables was made based on QQ‐plots and the Shapiro–Wilk test for normality. Differences in characteristics were tested with chi‐squared tests for categorical responses. The nonparametric Wilcoxon test was used for all continuous responses when they were not normally distributed. Parametric analyses of variance (ANOVAs) were used for exploratory investigations.

With the statistical function glm (binary response ~ risk factors, family = binomial [link = ‘logit’], data) in R, risk factors for LE were identified by means of logistic regressions.^14^, ^15^, ^16^, ^17^ In the logistic regression models, the dichotomous indicator (Y/N) of LE was used as response and the risk factors as independent variables. First, a starting set of potential risk factors was determined based on the univariate tests and the suggestions of the PCa specialist. After determination of the starting set of risk factors, a backward stepwise multiple logistic regression was used to select for the most parsimonious model. An alfa level of 10% was used for inclusion in the model. For all risk factors that stayed in the final model, odds ratios and their 95% confidence intervals were calculated. By comparing the residual deviances between models, likelihood ratio tests were used to tests whether the odds ratio was significantly different from one (log odds = 0). Using the same statistical model, the odds ratio of continuous independent variables could be estimated. The odds ratio is then for each increase of one unit of the risk factor.

## 3 RESULTS

### 3.1 Patients and tumour characteristics

After applying the inclusion and exclusion criteria, 500 unique patients were included in our dataset. A total of 301 patients (60%) underwent an ePLND. Preoperative patient and tumour characteristics are described in Table 1.

**TABLE 1:** Patient and tumour characteristics of all included patients and a comparison of these characteristics between patients with an ePLND and without during RARP.

Characteristics	Total population	ePLND −	ePLND +	Univariate statistic, df, *p* value
	*n* = 500	*n* = 199	*n* = 301	
Patient demographics				
Age (in years/median ± range)	66 [44–78]	65 [44–76]	66 [44–78]	*W* = 34 085, *p* < 0.009
BMI (in kg/m^2^/median ± range)	26.0 [18.1–39.4]	25.5 [18.7–39.4]	26.4 [18.1–38.7]	*W* = 33 292, *p* < 0.004
Smoking status (number/%)				Chi‐squared = 8.23, df = 2*, *p* < 0.02
Nonsmoker	129 (26%)	54 (27%)	75 (25%)	
Past smoker	218 (44%)	102 (52%)	116 (39%)	
Current smoker	58 (12%)	15 (8%)	43 (14%)	
Unknown	95 (19%)	28 (14%)	67 (22%)	
ASA score (number/%)				Chi‐squared = 0.16, df = 2*, *p* < 0.1
1	33 (7%)	14 (7%)	19 (6%)	
2	429 (86%)	169 (85%)	260 (86%)	
3	37 (7%)	14 (7%)	23 (8%)	
Unknown	1 (0%)	1 (1%)	/	
History of abdominal and/or vascular surgery (number/%)				Chi‐squared = 3.79, df = 1, *p* = 0.06
No	470 (94%)	182 (91%)	288 (95%)	
Yes	30 (6%)	17 (9%)	13 (4%)	
Tumour characteristics				
Staging PSA (in ng/mL/median/range)	7.8 [0.4–58]	6.7 [1.8–37]	8.8 [0.4–58]	*W* = 387 490, *p* < 0.00001
ISUP (number/%)				Chi‐squared = 209.68, df = 4, *p* < 0.00001
1	16 (3%)	14 (7%)	2 (1%)	
2	200 (40%)	148 (74%)	52 (17%)	
3	133 (27%)	31 (16%)	102 (34%)	
4	81 (16%)	6 (3%)	75 (25%)	
5	70 (14%)	‐	70 (23%)	
Clinical staging based on DRE (number/%)				Chi‐squared = 47.89, df = 4, *p* < 0.00001
cT1c	222 (44%)	126 (63%)	96 (32%)	
cT2	172 (34%)	71 (36%)	101 (34%)	
cT3a	92 (18%)	2 (1%)	90 (30%)	
cT3b	10 (2%)	‐	10 (3%)	
cT4	4 (1%)	‐	4 (1%)	
Risk group (number/%)				Chi‐squared = 205.46, df = 2, *p* < 0.00001
Low	8 (2%)	8 (4%)	‐	
Intermediate	262 (52%)	177 (89%)	85 (28%)	
High	230 (46%)	14 (7%)	216 (72%)	
Localized	128 (55%)	11 (78%)	117 (54%)	
Locally advanced	102 (44%)	3 (22%)	99 (46%)	

Abbreviations: ASA, American Society of Anesthesiologists; BMI, body mass index; ISUP, International Society of Urological Pathology; PSA, prostate‐specific‐antigen.

### 3.2 Intervention and post‐intervention details

Table 2 provides an overview of the intervention and post‐intervention details. A mean number of 23 (± 9) lymph nodes were removed in the ePLND group. Microscopic lymph node involvement was present in 13% of patients undergoing ePLND.

**TABLE 2:** Intervention and post‐intervention details of the ePLND and non‐ePLND subgroups.

Characteristics	Total population (*n* = 500)	Without ePLND (*n* = 199)	With ePLND (*n* = 301)	Univariate statistic, df, *p* value
Inguinal hernia repair (number/%)				Chi‐squared = 0.78, df = 1, *p* < 0.4
No	482 (96%)	194 (97%)	288 (96%)	
Yes	18 (4%)	5 (3%)	13 (4%)	
Number of nodes removed during RARP (mean ± std)	‐	‐	23 ± 9	
Pathological staging (number/%)				Chi squared = 18.6, df = 3, *p* < 0.0004
pT2	279 (56%)	145 (73%)	134 (44%)	
pT3a	173 (35%)	46 (23%)	127 (42%)	
pT3b	45 (9%)	7 (4%)	38 (13%)	
pT4	3 (1%)	1 (1%)	2 (1%)	
pN0	264 (53%)	2 (1%)	262 (87%)	
pN1	39 (8%)	‐	39 (13%)	
Adjuvant radiotherapy (number/%)	22 (4%)	0 (0%)	22 (7%)	
Prostate	6 (1%)	0 (0%)	6 (2%)	
Prostate + pelvic nodes	5 (1%)	0 (0%)	5 (2%)	
Prostate + pelvic + para‐aortic nodes	11 (2%)	0 (0%)	11 (4%)	
Salvage radiotherapy (number/%)	27 (5%)	8 (4%)	19 (6%)	
Prostate	22 (4%)	7 (4%)	15 (5%)	
Prostate + pelvic nodes	4 (1%)	1 (1%)	3 (1%)	
Prostate + pelvic + para‐aortic nodes	1 (0%)	0 (0%)	1 (0%)	
Total follow‐up time (in days/median/range)	540 (96–1484)	526 (100–1337)	554 (96–1484)	*W* = 31 517, *p* = 0.4

At the time of data extraction 49 (10%), patients had received adjuvant or salvage radiotherapy. In the ePLND group, 16 patients (5%) received adjuvant and 4 patients (1%) patients received salvage radiation therapy to the pelvic lymph nodes regions.

### 3.3 Incidence and characteristics of lymphedema

In this ePLND group, 78 out of the 301 patients (26%) developed secondary lymphedema. Most patients, 49/301 (16%), developed mild lymphedema (grade 1) which required no additional treatment in a specialized lymphedema clinic. In 29 patients (10%), the lymphedema was more pronounced (grade 2). These patients were referred to a specialized lymphedema clinic. Twenty‐six patients (9%) started treatment with decongestive lymphatic therapy consisting of compression stockings, skin care and exercises. In 35 out of the 78 (45%) patients, lymphedema appeared within 3 months after surgery. In 19 patients (24%), lymphedema appeared between 3 and 6 months after surgery, in 13 patients (17%) between 6 months and 1 year after surgery, in 9 patients (11%) between 1 and 2 years after surgery, whereas only in two patients (3%), lymphedema appeared 2 years after the surgery.

**FIGURE 1:**
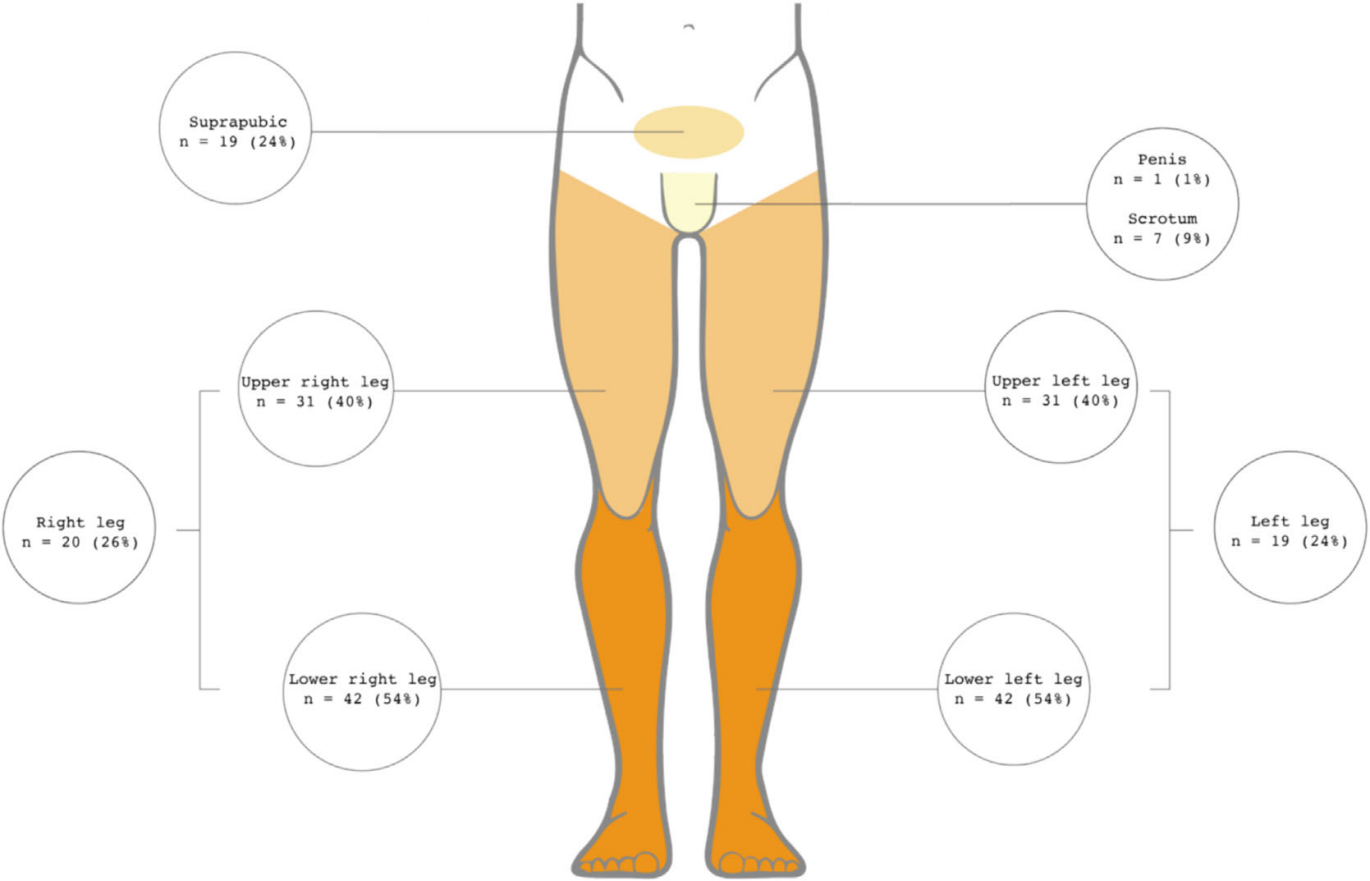
Distribution of lymphedema.

Details about the characteristics of secondary lymphedema are depicted in Figure 1. Lymphedema was most prevalent in the lower part of the legs (54% lower right leg and 54% lower left leg). Twenty patients had a swelling in the entire right leg (26%), and 19 patients had swelling in the entire left leg (24%). Eleven patients (14%) had lymphedema over the full length of both legs. Lymphedema in scrotum and penis were seldom reported (9% and 1%, respectively). Swelling of the suprapubic region was reported in 19 patients (24%).

The characteristics of ePLND patients with and without lymphedema are summarized in Table 3.

**TABLE 3:** Pre‐operative and post‐operative characteristics of patients with or without lymphedema after RARP with ePLND.

Characteristics	ePLND+ LE−, *n* = 223 (74%)	ePLND+ LE+, *n* = 78 (26%)	Univariate statistic, df, *p* value
Patient demographics			
Age (years/median + range)	67 [44–78]	66 [52–72]	*W* = 8429, *p* = 0.7
BMI (in kg/m^2^/median + range)	26.3 [18.1–38.7]	26.6 [20.8–38.5]	*W* = 7755.5, *p* = 0.2
Smoking status (number/%)			
Nonsmoker	56 (25%)	19 (24%)	Chi‐squared = 0.048, df = 2, *p* = 0.8^a^
Past smoker	87 (39%)	29 (37%)	
Current Smoker	30 (13%)	13 (17%)	
Unknown	50 (22%)	17 (22%)	
ASA score (number/%)			
1	13 (6%)	6 (8%)	Chi‐squared = 0.34, df = 2, *p* = 0.8
2	193 (87%)	66 (85%)	
3	17 (8%)	6 (8%)	
History of vascular surgery (number/%)			
No	213 (96%)	75 (96%)	Chi‐squared = 0.056, df = 1, *p* = 0.8
Yes	10 (4%)	3 (4%)	
Tumour characteristics			
Staging PSA (in ng/mL/median + range)	8.5 [0.9–58.0]	9.8 [0.4–55.0]	W = 8414, *p* = 0.7
ISUP (number/%)			
1	2 (1%)	0 (0%)	Chi‐squared = 4.16, df = 4, *p* = 0.4
2	37 (17%)	15 (19%)	
3	77 (35%)	25 (32%)	
4	60 (27%)	15 (19%)	
5	47 (21%)	23 (29%)	
Clinical staging (number/%)			
cT1c	72 (32%)	24 (31%)	Chi‐squared = 2.877, df = 4, *p* = 0.6
cT2	77 (35%)	24 (31%)	
cT3a	62 (28%)	28 (36%)	
cT3b	9 (4%)	1 (1%)	
cT4	3 (1%)	1 (1%)	
Risk group (number/%)			
Low	0 (0%)	0 (0%)	Chi‐squared = 0.08, df = 1^a^, *p* = 0.8
Intermediate	62 (28%)	23 (29%)	
High	161 (72%)	55 (71%)	
Localized	92 (41%)	25 (32%)	
Locally advanced	69 (31%)	30 (38%)	
Intervention & Post‐intervention Details			
Inguinal hernia repair (number/%)			Chi‐squared = 0.78, df = 1, *p* = 0.4
No	212 (95%)	76 (97%)	
Yes	11 (5%)	2 (3%)	
Console time (in min/median + range)	190 [120–360]	210 [100–310]	*W* = 6994, *p* < 0.01
Lateral border of the dissection (number/%)			Chi‐squared = 0.85, df = 1, *p* = 0.4
Extended	98 (44%)	39 (50%)	
Limited	125 (56%)	39 (50%)	
Number of nodes removed (median + range)	20 [2–66]	21 [9–73]	*W* = 7724.5, *p* = 0.14
Number of patients with positive nodes (number/%)	30 (13%)	9 (12%)	Chi‐squared = 0.9, df = 1, *p* = 0.7
Pathological staging (number/%)			
pT2	104 (47%)	30 (38%)	Chi‐squared = 2.16,
pT3a	90 (40%)	37 (47%)	df = 3, *p* = 0.5
pT3b	28 (13%)	10 (13%)	
pT4	1 (0%)	1 (1%)	
pN0	193 (87%)	69 (88%)	Chi‐squared = 0.19, df = 1, *p* = 0.7
pN1	30 (13%)	9 (12%)	
Total follow‐up time (in days/median+ range)	548 [96–1484]	594 [123–1472]	*W* = 7934, *p* = 0.2

Abbreviations: ASA, American Society of Anesthesiologists; BMI, body mass index; ePLND, extended pelvic lymph node dissection; ISUP, International Society of Urological Pathology; PSA, prostate‐specific‐antigen; RARP, robot‐assisted radical prostatectomy.

^a^Unknown not included in the analysis.

### 3.4 Potential risk factor investigation

BMI, pre‐ or perioperative inguinal hernia repair, number of nodes removed during RARP and adjuvant or salvage nodal radiotherapy were investigated as possible risk factors (Table 4).

**TABLE 4:** Multiple regression analysis in the ePLND+ population.

Characteristics	ePLND+ no lymphedema, *n* = 223 (74%)	ePLND+ lymphedema, *n* = 78 (26%)	Multiple logistic regression, *p* value, Wald statistics, sample size	Odds ratio, 95% CI
Patient demographics				
BMI (in kg/m^2^/median + range)	26.3 (18.1–38.7)	26.6 (20.8–38.5)	0.08 (LRT = 3.02, df = 1)	1.06 [0.99–1.15]
Intervention and post‐intervention details				
Inguinal hernia repair (number/%)				
No	212 (95%)	76 (97%)	0.2 (LRT = 1.67,df = 1)	0.38 [0.08–1.89]
Yes	11 (5%)	2 (3%)		
Number of nodes removed during RARP (median + range)	20 (2–66)	21 (9–73)	0.015 (LRT = 5.90, df = 1)	1.04 [1.01–1.07]
Neo‐adjuvant therapy (number/%)	15 (7%)	10 (13%)	0.2 (LRT = 1.6, df = 1)	1.80 [0.74–4.38]
Hormone therapy	15	10		
Adjuvant therapy or salvage therapy (number/%)	36 (16%)	10 (13%)		
Hormone therapy	34	9		
Radiotherapy	32	9	0.05 (LRT = 4, df = 1)	0.42 [0.17–1.0]
Combi				
	30	8		

Abbreviation: LRT, likelihood ratio test.

In the ePLND cohort (*n* = 301), the number of nodes removed (Wald test, *z* = 2.56, *n* = 301, *p* = 0.01, OR = 1.04, sd = 1.14) was the only factor that was significantly related to postoperative lymphedema development. For each additional lymph node removed during PLND, the odds of developing lymphedema increase with about 4%.

BMI and inguinal hernia repair were not significantly related according to the univariate regression. To evaluate the contribution of these last three factors in the presence of the other significant risk factors, we also performed multiple regression tests. In the multiple regression model, these risk factors remained not significantly related to the lymphedema findings.

## 4 DISCUSSION

In our analysis, one in four patients (26%) who underwent ePLND developed lymphedema persisting or presenting 6 weeks after surgery. This incidence of lymphedema in our cohort is high compared to other studies.^2^, ^5^, ^18^ In a recent systematic review, the incidence of secondary LE after radical prostatectomy with ePLND ranged from 0 to 14%.^7^

The higher incidence of lymphedema in our study population compared to other literature can be attributed to several factors. First, the surgical technique and extent of the lymph node dissection may affect the incidence of lymphedema. In our cohort, the genitofemoral nerve was used as the lateral border of our eLND template. Although most studies reporting LE rates do not describe the specific surgical technique for LND, extending the eLND template to the genitofermoral nerve may indeed increase the risk of lower limb lymphedema.^19^ In line herewith, the mean number of lymph nodes removed (23 ± 9) is higher than reported in other contemporary series, suggesting a more extended lymph node dissection, which may have contributed to increased postoperative lymphedema.^20^

Second, the prospective use of structured eCRFs with a dedicated section for lymphedema may have led to increased attention to the issue during follow‐up visits. In contrast to more invalidating complications like urinary incontinence and erectile dysfunction, awareness for secondary lymphedema in literature and clinical practice is low. Although 26% of patients developed some degree of lymphedema, only 10% developed stage 2 lymphedema, which is in line with reported lymphedema rates in literature. It is noteworthy to highlight the LAPPRO trial, which is the only study reporting the prospective assessment of patient‐reported groin and leg swelling.^9^ In this study, 16% of patients reported little swelling, whereas 13.7% of patients reported moderate to severe swelling. These numbers are comparable to our cohort, where respectively 16% and 10% of grade 1 (mild) and 2 grade (moderate) lymphedema were reported.

Third, incidence rates of lymphedema may vary among different studies because of the lack of standardized diagnostic criteria for lymphedema, resulting in discrepancies between different centres. In our study, the presence of swelling of the legs, genital or suprapubic area was recorded, based on the patient's complaints and the health care professional's clinical judgement. Only in patients with grade 2 lymphedema, referred to our lymphedema clinic, circumferential measurements of the limb were performed. Similarly, the LAPPRO trial used a questionnaire to assess patient and staff‐reported lymphedema 3 months after surgery, without the use of standardized measuring tools.^9^

In addition to the lymph node dissection procedure itself, our study identified the number of dissected lymph nodes as a significant contributing factor to the risk of developing lymphedema. Comparable findings have been reported in breast cancer research.^21^ In contrast, elevated body mass index and adjuvant therapy were no significant risk factors in this prostate cancer dataset. Of the patients who developed lymphedema, only a third had grade 2 lymphedema and was referred to a specialized lymphedema centre. Twenty‐six (9%) patients undergoing ePLND received decongestive lymphatic therapy. Thus, although about one in four patients reports some form of lymphedema; only one in 10 will need additional treatment. To the best of our knowledge, no other data about the rate of decongestive lymphatic therapy are available in this setting.

This study has a number of limitations. Although the database was constructed prospectively, using structured CRFs, this was a single‐centre retrospective analysis with its inherent biases. The lack of standardized diagnostic criteria for lower limb and midline lymphedema may result in reporting bias (both over and underreporting) of lymphedema, especially grade 1 lymphedema for which no specific treatment was needed. We did not use a standardized questionnaire to assess patient‐reported lymphedema and the impact on quality of life. However, we investigated the impact of different potential risk factors, patients who received ePLND showed significant variation in baseline characteristics such as age, BMI and smoking status, PSA, ISUP, clinical staging and risk group compared to those without ePLND, which could lead to a biased comparison. Finally, in our risk factor analysis, we did not focus on the relation between laterality of the removed lymph nodes and the occurrence of lymphedema. Nevertheless, the study provides a more detailed estimate of the incidence and characteristics of lymphedema, underlying the clinical relevance of this medical condition.

Although ePLND is still considered the most accurate staging method for detecting pathologic lymph node involvement, the oncological benefits remain unproven.^2^ Moreover, this procedure not only increases the risk of short‐term postoperative complications, but also of long‐term lymphedema, necessitating decongestive lymphatic therapy, as demonstrated in this manuscript. With the advent of advanced imaging modalities, such as PSMA PET CT/MRI and image guided surgery, the role of an ePLND is further scrutinized.^22^, ^23^ Patients should therefore be counselled on the benefits of better staging versus the harms of this procedure.

## 5 CONCLUSION

In this cohort study, approximately one in four patients who underwent RARP with ePLND developed lower limb and/or midline oedema, whereas one in 10 patients started decongestive lymphatic therapy. The number of lymph nodes removed was identified as a risk factor for secondary lymphedema. These findings provide crucial information for patient counselling on the risks associated with extended pelvic lymph node dissection (ePLND), particularly highlighting lymphedema as a potential complication.

## AUTHOR CONTRIBUTIONS

*Conceptualization*: Wouter Everaerts. *Methodology*: Andries Clinckaert, Luc Bijnens, Steven Joinau and Wouter Everaerts. *Writing—original draft preparation*: Andries Clinckaert, Laura Ysenbaardt, Annabel Bijnens and Wouter Everaerts. *Writing—review and editing*: Andries Clinckaert, Charlotte Van Calster, Inge Geraerts, Steven Joniau, Nele Devoogdt and Wouter Everaerts. *Visualization*: Andries Clinckaert, Laura Ysenbaardt and Annabel Bijnens. Supervision: Steven Joniau and Wouter Everaerts. All authors have read and agreed to the published version of the manuscript.

## CONFLICT OF INTEREST STATEMENT

In accordance with the standards for transparent disclosure, the following conflicts of interest are declared: Steven Joniau and Wouter Everaerts hold the position of Senior Clinical Researcher at the Research Foundation – Flanders (FWO); Luc Bijnens holds stocks in various pharmaceutical companies not leading to a financial interest related to the subject matter of this research. All authors affirm their commitment to upholding objectivity and integrity in the research process, ensuring that these potential conflicts do not compromise the quality or impartiality of the findings presented.

## References

[1] Hermsen R , Wedick EBC , Vinken MJM , van Kalmthout LWM , Küsters‐Vandevelde HVN , Wijers CHW , et al. Lymph node staging with fluorine‐18 prostate specific membrane antigen 1007‐positron emission tomography/computed tomography in newly diagnosed intermediate‐ to high‐risk prostate cancer using histopathological evaluation of extended pelvic node dissection as reference. Eur J Nucl Med Mol Imaging. 2022;49(11):3929–3937. 10.1007/s00259-022-05827-4 35543733

[2] Fossati N , Willemse PPM , van T , van R , Yuan C , Briers E , et al. The benefits and harms of different extents of lymph node dissection during radical prostatectomy for prostate cancer: a systematic review. Eur Urol. 2017;72(1):84–109. 10.1016/j.eururo.2016.12.003 28126351

[3] Executive Committee of the International Society of Lymphology . The diagnosis and treatment of peripheral lymphedema: 2020 consensus document of the International Society of Lymphology. Lymphology. 2020;53(1):3–19. 10.2458/lymph.4649 32521126

[4] Liu F , Liu N , Wang L , Chen J , Han L , Yu Z , et al. Treatment of secondary lower limb lymphedema after gynecologic cancer with complex decongestive therapy. Lymphology. 2022;54(3):122–132. 10.2458/LYMPH.4786 34929073

[5] Keegan KA , Cookson MS . Complications of pelvic lymph node dissection for prostate cancer. Curr Urol Rep. 2011;12(3):203–208. 10.1007/S11934-011-0179-Z 21394597

[6] Rasmusson E , Gunnlaugsson A , Blom R , Björk‐Eriksson T , Nilsson P , Ahlgen G , et al. Low rate of lymphedema after extended pelvic lymphadenectomy followed by pelvic irradiation of node‐positive prostate cancer. Radiat Oncol. 2013;8(1):271. 10.1186/1748-717X-8-271 24252686 PMC3842657

[7] Clinckaert A , Callens K , Cooreman A , Bijnens A , Moris L , van Calster C , et al. The prevalence of lower limb and genital lymphedema after prostate cancer treatment: a systematic review. Cancers (Basel). 2022;14(22):5667. 10.3390/cancers14225667 36428759 PMC9688147

[8] Cormier JN , Askew RL , Mungovan KS , Xing Y , Ross MI , Armer JM . Lymphedema beyond breast cancer. Cancer. 2010;116(22):5138–5149. 10.1002/cncr.25458 20665892

[9] Carlsson S , Bottai M , Lantz A , Bjartell A , Hugosson J , Steineck G , et al. Lymph swelling after radical prostatectomy and pelvic lymph node dissection. BJU Int. 2022;129(6):695–698. 10.1111/bju.15702 35132753 PMC9313832

[10] Akand M , Muilwijk T , Cornelissen J , van Bruwaene S , Vander Eeckt K , Baekelandt F , et al. Development of a prospective data registry system for non‐muscle‐invasive bladder cancer patients incorporated in the electronic patient file system. Front Oncol. 2019;9:1402. 10.3389/fonc.2019.01402 31921659 PMC6917611

[11] Rockson SG . Lymphedema after breast cancer treatment. N Engl J Med. 2018;379(20):1937–1944. 10.1056/NEJMcp1803290 30428297

[12] Lindqvist E , Wedin M , Fredrikson M , Kjølhede P . Lymphedema after treatment for endometrial cancer − a review of prevalence and risk factors. Eur J Obstet Gynecol Reprod Biol. 2017;211:112–121. 10.1016/j.ejogrb.2017.02.021 28242470

[13] Huang J , Yu N , Wang X , Long X . Incidence of lower limb lymphedema after vulvar cancer: a systematic review and meta‐analysis. Medicine (United States). 2017;96(46):1–6. 10.1097/MD.0000000000008722 PMC5704859 29145314

[14] Molenberghs G , Verbeke G . Models for discrete longitudinal data 3rd ed. Springer; 2012.

[15] Verbeke G . Linear Mixed Models for Longitudinal Data. In: Linear mixed models in practice 126 Springer New York; 1997. p. 63–153.

[16] Neter J and KMH and NCJ and WW and others . Applied linear statistical models. Irwin Chicago. Published online 1996.

[17] Agresti A . An Introduction to categorical data analysis 2nd ed. Wiley‐Interscience; 2007.

[18] Musch M , Klevecka V , Roggenbuck U , Kroepfl D . Complications of pelvic lymphadenectomy in 1,380 patients undergoing radical Retropubic prostatectomy between 1993 and 2006. J Urol. 2008;179(3):923–929. 10.1016/J.JURO.2007.10.072 18207170

[19] Williams SB , Bozkurt Y , Achim M , Achim G , Davis JW . Sequencing robot‐assisted extended pelvic lymph node dissection prior to radical prostatectomy: a step‐by‐step guide to exposure and efficiency. BJU Int. 2016;117(1):192–198. 10.1111/BJU.13228 26190197

[20] May M , Gilfrich C , Bründl J , Ubrig B , Wagner JR , Gloger S , et al. Impact of peritoneal interposition flap on patients undergoing robot‐assisted radical prostatectomy and pelvic lymph node dissection: a systematic review and meta‐analysis of randomized controlled trials. Eur Urol Focus. 2024;10(1):80–89. 10.1016/J.EUF.2023.07.007 37541915

[21] DiSipio T , Rye S , Newman B , Hayes S . Incidence of unilateral arm lymphoedema after breast cancer: a systematic review and meta‐analysis. Lancet Oncol. 2013;14(6):500–515. 10.1016/S1470-2045(13)70076-7 23540561

[22] Hofman MS , Lawrentschuk N , Francis RJ , Tang C , Vela I , Thomas P , et al. Prostate‐specific membrane antigen PET‐CT in patients with high‐risk prostate cancer before curative‐intent surgery or radiotherapy (proPSMA): a prospective, randomised, multicentre study. Lancet. 2020;395(10231):1208–1216. 10.1016/S0140-6736(20)30314-7 32209449

[23] Everaerts W , Walz J , Abascal Junquera JM , Goffin K , Grootendorst MR , van't Klooster K , et al. A multicentre clinical trial evaluating a drop‐in gamma probe for minimally invasive sentinel lymph node dissection in prostate cancer. Eur Urol Focus. 2024;10(1):32–40. 10.1016/j.euf.2023.07.001 37495459

